# Emotion recognition and extraversion of medical students interact to predict their empathic communication perceived by simulated patients

**DOI:** 10.1186/s12909-018-1342-8

**Published:** 2018-10-11

**Authors:** Teresa Schreckenbach, Falk Ochsendorf, Jasmina Sterz, Miriam Rüsseler, Wolf Otto Bechstein, Bernd Bender, Myriam N. Bechtoldt

**Affiliations:** 1 Department of General and Visceral Surgery, Frankfurt University Hospital, Goethe-University Frankfurt/Main, Theodor-Stern-Kai 7, 60590 Frankfurt/Main, Germany; 2 Department of Dermatology, Frankfurt University Hospital, Frankfurt/Main, Germany; 3 Department of Trauma Surgery, Frankfurt University Hospital, Frankfurt/Main, Germany; 4 Department of Management & Economics, EBS University of Business and Law, Oestrich-Winkel, Germany

**Keywords:** Big five, Emotional recognition, Personality, Simulated patients, Medical education

## Abstract

**Background:**

This study assessed the impact of medical students’ emotion recognition ability and extraversion on their empathic communication, as perceived by simulated patients in a training context.

**Methods:**

This study used a crossed-effect data structure and examined 245 students in their fourth year of medical school. The students’ personality traits were assessed based on a self-assessment questionnaire of the short form of the Big Five Inventory; their emotion recognition ability was measured using a performance test (Diagnostic Analysis of Nonverbal Accuracy-2, Adult Facial Expressions). Simulated patients evaluated the medical students’ empathic communication.

**Results:**

Students with a combination of high emotion recognition ability and extraversion received more positive ratings from simulated patients than their fellow students with a combination of emotion recognition ability and low extraversion. The main effects of emotion recognition or extraversion were not sufficient to yield similar effects. There were no other effects related to the remaining Big Five variables.

**Conclusions:**

The results support the hypothesis that to build rapport with patients, medical staff need to combine emotional capabilities with a dispositional interest in interpersonal encounters.

## Background

Despite the increasing use of technology in medicine, face-to-face communication in the doctor-patient relationship remains vital for successful treatment outcomes [1, 2]. This is also reflected in the increasing number of papers assessing medical students’ verbal and non-verbal communication skills [3–5]. People communicate a large amount of information nonverbally, which means that successful communication includes the ability to decode others’ facial expressions, body postures, and tone of voice. This ability is part of emotional intelligence (EI), which is defined as the ability to perceive one’s own feelings and those of others, as well as to understand and influence those feelings [6]. According to Mayer and colleagues, EI consists of four components: (1) perceiving emotion (emotion recognition), (2) facilitating thought with emotion, (3) understanding emotion, and (4) managing emotion [7]. Emotion recognition is one part of EI that comprises the ability to distinguish different body postures, facial expressions, and gestures [8]. Therefore, emotion recognition is at the core of EI and is a prerequisite for successful social interactions [9].

Notably, whether individuals use their ability of emotion recognition for prosocial behavior depends on their individual personality traits [10]. There are different theories for personality traits. One of the most used and well-approved theories is the Five-Factor model, also known as the Big Five Inventory [11]. In this model, there are five broad dimensions of personality traits: (1) openness, (2) conscientiousness, (3) extraversion, (4) agreeableness, and (5) neuroticism [12]. People with high openness have a vivid imagination, love art, and prefer variety. People with high conscientiousness are reliable, hard-working, and deliberate, whereas extraverted individuals are characterized by activity, warmth, friendliness, and positive emotions [12]. Agreeableness refers to trustworthiness, altruism, and modesty [12]. People scoring high on neuroticism are characterized as vulnerable, impulsive, and anxious.

Personality traits are usually determined by self-assessment questionnaires, a method well-accepted in research [13]. In fact, personality traits were shown to relate to professional preferences of medical students [14]. For example, the choice of medical students for a specific medical field after finishing medical school also seems to depend on their personality, as students with higher scores in extraversion, agreeableness, and openness appear to find their ways into departments with greater patient contact [15]. On the other hand, tests of personality traits were not sufficient to predict medical students’ exit performance in medical school [16]. Previous research also had a closer look at the association between physicians’ dispositional traits and their verbal and nonverbal communication skills [17]. Usually, patient-centered communication is rated by patients and observers as the best communication style in these relationships [18]. This consists of caring and decision sharing and is known to lead to higher patient satisfaction in most cases. In particular, nonverbal sensitivity, including the ability to recognize the feelings and emotional states of the interaction partner, has been shown to have a high impact on the quality of the doctor-patient relationship [17]. For example, Hall and colleagues have shown that high nonverbal sensitivity was associated with more empathy in interactions with student-simulated patients [19]. Typically, women, including female medical students, are known to have higher levels of self-report empathy and compassion [20, 21], which might affect communication styles with patients. Indeed, Berg and colleagues matched students’ self-reported empathy with simulated patients’ (SPs) perception of empathy [22] and found a significant correlation between self-reports and patients’ perception of empathy. However, the authors were unable to make a direct match between students’ self-assessed empathy and SPs perceived empathy. Rather, they conducted an overall comparison. Therefore, it is still unclear whether self-reported empathy positively affects patient satisfaction on the dyadic level. Furthermore, Baez and colleagues suspected women’s higher scores in empathy self-assessment forms to be biased by gender-role stereotypes in self-assessments [20]: Women might ascribe themselves more empathy simply because society expects them to do so [23]. However, there is some evidence that female medical students’ higher scores in empathy are not only an artifact of self-assessment because they also achieved higher scores in observer-rated interactions with simulated patients [24, 25]. Nevertheless, such findings have been inconsistent [26].

Therefore, more research is necessary, including an objective assessment of medical professionals’ empathic communication in interactions with patients. Typically, this is done with the help of simulated patients (SPs). SPs are lay people trained to act as a patient in a special situation [27]. SPs were shown to be reliable assessors in medical exams [23, 28]. Also, Wright and colleagues found a high inter-rater correlation when SPs assessed medical students’ empathy [29]. Although several studies have investigated perceived empathic communication between students and SPs [30, 31], only a few studies have dealt with the dispositional precursors of empathic communications interaction, including emotion recognition abilities and non-performance related personality traits like the Big Five [16, 22]. Due to the inconclusive results of previous research, the combined influence of medical students’ dispositional traits and emotion recognition ability on SPs perceived empathic communication is still unclear.

There is evidence from other areas that, in social interactions, the senders’ personality traits, in combination with a high ability of emotion recognition, affect the targets’ perception of empathic communication [32]. In particular, employees rated managers’ leadership styles as more transformational if managers combined extraversion with emotion recognition [10]. Transformational leadership is defined as a highly successful and active form of leadership in which leaders are considerate, motivating, and closely engaged with followers, which is similar to empathic communication [10, 33]. Therefore, emotion recognition and extraversion of physicians in patient care might be equally relevant to elicit positive reactions from patients. For example, in communications with their patients, physicians need to take the role of leaders, but they also need to collaborate with patients and be considerate to motivate them for treatment [34]. Therefore, traits and abilities similar to those in management might be used successfully to communicate with patients. More precisely, patients might evaluate physicians’ communication more positively and as more empathic if the latter combined emotion recognition ability with extraversion. Extraverted physicians can be expected to display positive emotions and take the lead in communications with their patients [35]; thanks to emotion recognition ability, they would remain sensitive and proactive to patients’ nonverbal signals.

This study aimed to contribute to addressing this research gap. In particular, we hypothesized that medical students combining high emotion recognition with extraversion would receive more positive evaluations from SPs concerning their empathic communication. We used a special setting with trained SPs during an objective structured clinical examination (OSCE) in surgery with medical students who had undergone practical training in basic skills and completed an internship on a surgical ward [36].

## Methods

### Participants

All of the medical students in their fourth year undergoing surgical OSCE in 2014/2015 at the Medical Clinics of the University Frankfurt/Main were asked to participate in the study. Only students who provided written consent were included. All of the students had undergone 1 week of practical training in basic skills, such as obtaining blood, conducting a physical examination, taking a history, and discussing informed medical consent. A focus of this training was how to correctly communicate with patients and practice correct interpersonal behavior. Afterward, to further develop their skills, all of the students participated in an internship on a surgical ward for 2 weeks.

A total of 367 students were asked to participate in the study, and 245 students (67%) agreed and returned their self-assessment forms. The mean age of the cohort was 23.25 ± 2.48 years, and 155 of the students (63%) were female (Table 1). There were 62 cases in which the data could not be used for analysis due to missing or illegibly written text. Reasons for this, for example, were missing parts of the self-assessment form or making two crosses on the same question. The remaining 183 students were included in hypothesis testing.

**Table 1:** Descriptive statistics of student and simulated patients demographics, self-assessment of the Big Five inventory and empathic communication as perceived by SPs

		*M*	*SD*	1	2	3	4	5	6	7	8	9	10
1	Age	23.25	2.48										
2	Gender	1.36	.48	.048									
3	Age (SP)	60.94	5.15	−.118	−.131								
4	Gender (SP)	1.45	.27	−.090	.121	.078							
5	Emotion Recognition	19.05	4.00	.007	−.153*	−.022	−.116						
6	Extraversion	3.68	.84	.031	−.104	.073	.028	.123					
7	Openness	4.07	.71	−.012	−.099	.036	.081	.055	.175*				
8	Conscientiousness	3.90	.59	.024	−.209**	.059	.153*	−.003	.215**	.048			
9	Agreeableness	3.11	.77	.060	−.339**	.081	.071	−.078	.232**	.099	.271**		
10	Neuroticism	2.98	.86	−.123	−.209**	−.057	−.045	.021	−.265**	.087	−.102	−.071	
11	Empathic Communication^a^	3.78	.54	.030	−.160*	−.155*	−.261**	.139	.038	−.197**	−.006	−.033	.071

Note. *N* = 183; ^a^Evaluated by SP; *p ≤ .05; **p ≤ .01

*M* mean; *SD* standard deviation; *SP* simulated patients

### Simulated patients

A total of 37 SPs participated in the study. The median age of participants was 59 years (range, 25–76 years). Most (62.6%) of the SPs were female. All of the SPs were trained for their roles by the SP trainer and had their individual case vignettes.

The SPs received verbal and written information about the study and were able to ask questions about the measurement prior to the start of the OSCE. Also, the SPs were informed that their assessments had no negative or positive effects on the OSCE results of the students.

### Data collection

Students were asked to complete a self-assessment questionnaire during a 1-week course called “Training of Practical Skills”. After the course, students participated in a 2-week practical course in surgical wards in various clinics. Assessment of the course was executed as an OSCE. Simulated patients rated students’ performance (i.e., their understanding and level of care) immediately after the examination. Every student had to pass through four stations with SPs, with three possible simulated situations: (1) a medical informed consent discussion prior to surgery, (2) obtaining a medical history, and (3) conducting a physical examination. All OSCE stations took 5 min, and 1 min was allotted to move between the stations.

To make sure that the students’ self-assessment forms and the SPs’ assessment forms could be correlated, all of the students filled in a special code in the self-assessment forms and received stickers for writing down the code on the patient evaluation forms, which they gave to the SPs. The code was anonymous but unique, and there was no possibility of drawing conclusions about the students from the code.

The study was conducted according to the ethical principles of the Helsinki Declaration (Ethical Principles for Medical Research Involving Human Subjects), and no ethical concerns were raised by the ethics committee of the medical faculty of the Goethe University, Frankfurt, Germany.

### Measures

#### Extraversion

To measure extraversion, we used the German translation of the Big Five Inventory (BFI)-short (BFI-K) questionnaire [37], which was established in 2005 as a short version of the BFI [13]. The items were rated on a five-point Likert scale ranging from 1 = strongly disagree to 5 = strongly agree [37]. Internal consistency of extraversion (four items) was α = 0.79.

#### The remaining big-five traits

Although the focus of this study was on extraversion, we additionally evaluated the remaining Big Five variables for exploratory reasons. Their internal consistencies were α = 0.74 (openness, five items), 0.63 (conscientiousness, four items), 0.58 (agreeableness, four items), and 0.77 (neuroticism, four items). Thus, the internal consistencies of conscientiousness and agreeableness were low. Table 2 shows the BFI-short-version.

**Table 2:** Big-five-inventory short version (BFI-short) [37]

I see myself as someone who ....	
1.	...is outgoing, sociable.
2.	...generates a lot of enthusiasm.
3.	...tends to be quiet.
4.	...is reserved.
5.	...is generally trusting.
6.	...tends to find fault with others.
7.	...can be cold and aloof.
8.	...is sometimes rude to others.
9.	...does things efficiently.
10.	...does a thorough job.
11.	...makes plans and follows through with them.
12.	...tends to be lazy.
13.	...gets nervous easily.
14.	...worries a lot.
15.	...is depressed, blue.
16.	...is relaxed, handles stress well.
17.	...values artistic, aesthetic experiences.
18.	...is curious about many different things.
19.	...has an active imagination.
20.	...is ingenious, a deep thinker.
21.	...has few artistic interests.

#### Emotion recognition

To measure emotion recognition, we used the Diagnostic Analysis of Nonverbal Accuracy-2, Adult Facial Expressions (DANVA-2-AF), which consists of 24 photographs of adult faces showing an equal number of happy, sad, angry, and fearful facial expressions of high or low intensity [38]. The students had to decide which facial expressions were shown. The answers were judged as right or wrong. A higher score resulted from more faces being correctly recognized. The DANVA-2-AF is widely used in field research [39–41]. All of the students received a set of all 24 color printed photographs and an answer sheet. The DANVA-2-AF has been shown to be a reliable and valid measure of emotion recognition [42]. Internal consistency was α = 0.84. Students were divided in low (− 1 standard deviation (SD)) and high (+ 1 SD) ability of emotion recognition.

### Perceived empathic communication

To measure simulated patients’ perceived empathic communication, we used the 10-item Consultation and Relational Empathy (CARE) scale developed by Mercer and colleagues [43]. This scale measures whether or not a doctor exhibits an understanding of a patient’s problems. In a pilot study with 95 students and 10 SPs, we measured whether CARE items applied to a study context in which medical students interacted with SPs for 5 min to elicit diagnostic information. Simulated patients considered the following five items appropriate: (1) Making you feel at ease, (2) Letting you tell your “story,” (3) Truly listening, (4) Showing care and compassion, and (5) Being interested in you as a whole person. Items were rated on a five-point scale (1 = poor; 5 = excellent). Internal consistency was excellent (α = 0.91) and equal to that of the full scale (α = 0.93).

### Statistical analysis

**Fig. 1:**
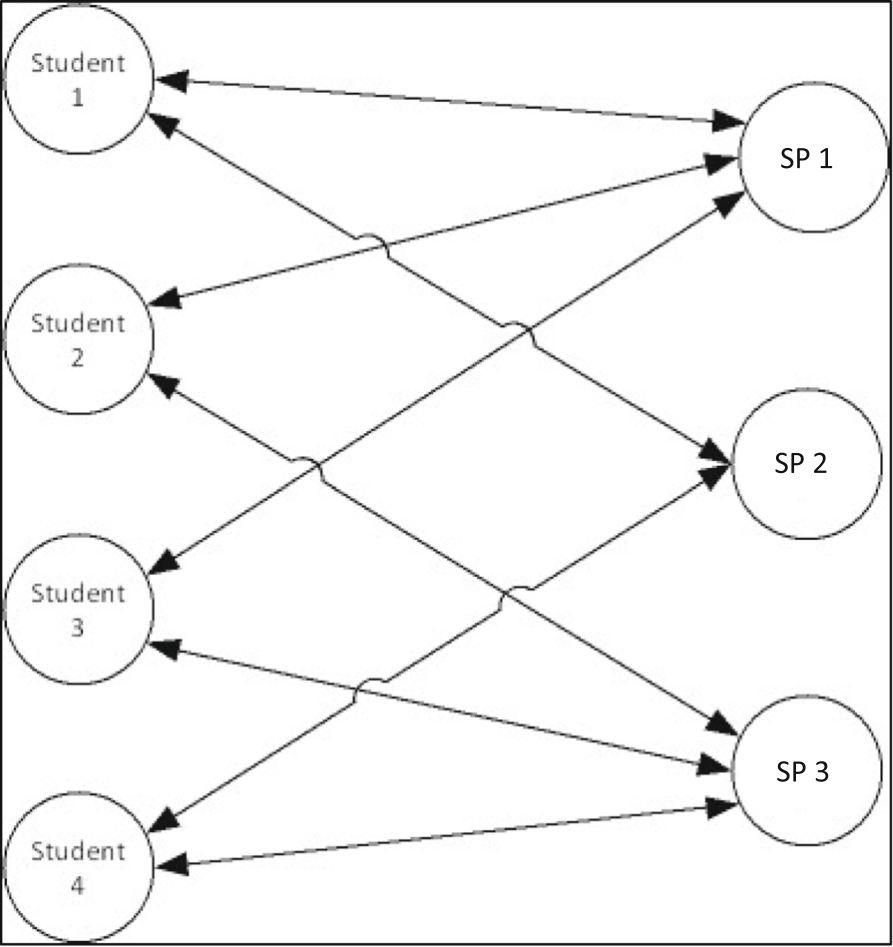
Model of the data structure in the study. Students and simulated patients (SP) are crossed with one another

The design of the study implied a crossed-effect data structure. For each student, there were multiple behavior ratings by SPs, suggesting that patients were “nested” among students. Given that each SP evaluated a subsample of students, students were also “nested” within SPs. Figure 1 displays the data structure. Therefore, we analyzed linear mixed models with crossed random effects. These models enable potential correlations of the repeated observations (the patient satisfaction ratings) with each level of these crossed random factors to be modeled simultaneously [44]. For example, students might truly differ in their behaviors towards SPs, which could result in between-student variance in patient satisfaction scores. However, at the same time, the SPs might be another source of variance in students’ behavior ratings: SPs might have different standards for what they consider adequate behavior, resulting in between-patient variance in satisfaction with students’ performance. Each SP judged a subsample of students but not the entire sample of students, which means that differences in patients’ satisfaction scores might derive from SPs’ individual evaluation standards rather than students’ behaviors. The crossed random effects models take into account the intertwined levels of students and SPs. The analyses were performed with the lme4-package in R [45].

## Results

Table 1 reports descriptive statistics and intercorrelations of all variables. Neither students’ emotion recognition nor extraversion covaried with SPs perceived empathic communication. However, there were significant negative correlations between students’ gender and empathic communication (− 0.16), suggesting that male students were perceived as communicating less empathically, as well as between students’ openness and their empathic communication as perceived by the SPs (− 0.20). Furthermore, SPs of higher age perceived students as communicating less empathically (− 0.16); male SPs shared this opinion (− 0.26).

### Hypothesis testing

We first analyzed a null model including the two crossed data levels of students and SPs to assess the intraclass correlations (ICCs) for patient satisfaction. The ICC denotes the amount of variance in patient satisfaction scores explained by differences in the grouping variables. Given the crossed data structure, we analyzed ICCs for students and SPs as grouping variables separately. The ICC with students as a grouping variable was 0.12, and that with SPs as a grouping variable was 0.37. Therefore, 12% of the variance in patient satisfaction scores could be explained by differences between students, and 37% could be explained by differences between SPs themselves. The SPs differed considerably in their evaluations of students’ behavior.

The following analyses test if students’ emotion recognition and extraversion interact to predict how SPs rate students’ behavior. In each model, we allowed individual students’ scores to deviate from model-predicted mean scores of perceived empathic communication. Furthermore, we allowed the impact of emotion recognition and extraversion on perceived empathic communication scores to vary individually. Technically, this translates into modeling random intercepts and random slopes. We also allowed individual SPs to deviate from their model-predicted evaluation of perceived empathic communication, which translated into modeling random intercepts for their ratings. We controlled for gender and age of both the students and SPs since demographic variables might unconsciously affect the SPs’ ratings.

**Fig. 2:**
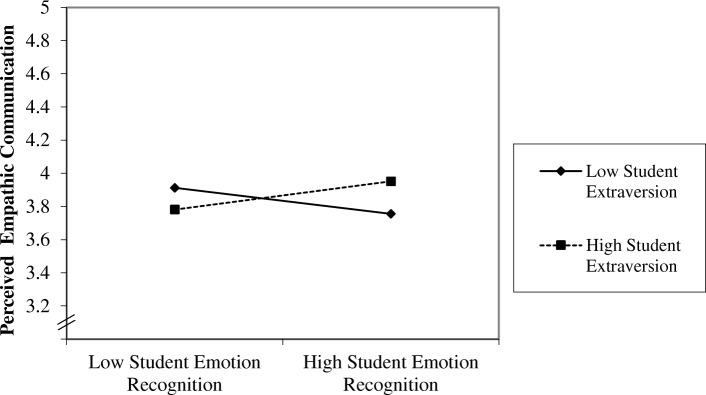
Interaction of students’ emotion recognition and extraversion (low / high = − / + 1 SD) on simulated patients (SP) perceived empathic communication

According to the hypothesis, we predicted that SPs would rate interactions with students more positively if students combined emotion recognition with extraversion. The results are listed in Table 3. The fixed effects represent the average results across all students, irrespective of individual deviations. Among the control variables, there was a main effect for SPs’ gender, indicating that male SPs evaluated students’ empathic communication less positively overall. The table shows that neither the main effect of student’s emotion recognition nor extraversion predicted simulated patients’ perceived empathic communication. However, as predicted, the interaction of emotion recognition and extraversion did so: Students combining emotion recognition with extraversion received higher performance ratings than their fellow students combining emotion recognition with low extraversion. The interaction is shown in Fig. 2. Therefore, the hypothesis is supported: Students’ emotion recognition and extraversion interact to predict perceived empathic communication.

**Table 3:** Parameter estimates for crossed random effects model of student empathic behavior (judged by simulated patients) depending on students’ emotion recognition and extraversion

Fixed effects	Estimate	*SE*	*t*	*p*
Intercept	3.85	0.14	27.62	< 0.001**
Emotion Recognition (ER)	< 0.01	0.01	0.14	0.889
Extraversion	0.02	0.04	0.64	0.523
ER x Extraversion	0.03	0.01	2.32	0.021*
*Control Variables*				
Age	−0.02	0.02	−0.95	0.342
Gender^a^	− 0.05	0.08	− 0.61	0.546
SP Age	− 0.01	0.01	− 0.70	0.489
SP Gender^a^	−0.42	0.19	−2.21	0.035*

^a^0 = female, 1 = male; *p ≤ .05; **p ≤ .01

*SE* standard errror; *t* t-value *ER* emotion recognition

For exploratory reasons, we also analyzed the remaining Big Five variables (Table 4). There were neither significant main effects of the remaining Big Five variables nor did they interact with emotion recognition. As before, there was a negative main effect of simulated patients’ gender, suggesting that male simulated patients’ perceived students’ communication behavior as less empathic.

**Table 4:** Parameter estimates for crossed random effects model of student empathic behavior (judged by simulated patients) depending on students’ emotion recognition and the remaining Big Five variables

Fixed effects	Estimate	*SE*	*t*	*p*	Estimate	*SE*	*t*	*p*	Estimate	*SE*	*t*	*p*	Estimate	*SE*	*t*	*p*
Intercept	3.87	0.14	27.87	< 0.001	3.86	0.14	27.59	<.001	3.87	0.14	27.64	<.001	3.88	0.14	27.91	< 0.001
Emotion Recognition (ER)	−0.01	0.01	−0.67	0.51	−0.01	0.01	−0.47	0.64	0.00	0.01	−0.39	0.69	0.00	0.01	−0.27	0.79
Agreeableness	−0.04	0.05	−0.71	0.48												
ER x Agreeableness	0.01	0.01	0.64	0.53												
Neuroticism					0.02	0.05	0.39	0.70								
ER x Neuroticism					−0.01	0.02	−0.90	0.37								
Conscientiousness									0.01	0.07	0.17	0.86				
ER x Conscientiousness									0.01	0.02	0.46	0.65				
Openness													−0.11	0.05	−2.21	0.03
ER x Openness													0.00	0.01	0.07	0.94
*Control Variables*																
Age	−0.01	0.02	−0.92	0.36	−0.01	0.02	−0.89	0.37	−0.01	0.02	−0.91	0.36	−0.01	0.02	−0.92	0.36
Gender^a^	−0.06	0.09	−0.75	0.45	−0.04	0.08	−0.45	0.66	−0.05	0.08	−0.60	0.55	−0.07	0.08	−0.83	0.41
SP Age^a,b^	−0.01	0.01	−0.69	0.50	−0.01	0.01	−0.65	0.52	−0.01	0.01	−0.66	0.51	−0.01	0.01	−0.67	0.51
SP Gender^a,b^	−0.43	0.19	−2.31	0.03*	−0.44	0.19	− 2.31	0.03*	−0.44	0.19	−2.31	0.03*	−0.44	0.19	−2.33	0.03*

^a^0 = female, 1 = male; *p ≤ .05; **p ≤ .01. ^b^SP = simulated patient

*SE* standard errror; *t* t-value, *ER* emotion recognition

## Discussion

The goal of this study was to examine the influence of emotion recognition and extraversion of medical students on SPs perception of empathic communication. In a standardized context requiring medical students to interview SPs about their symptoms, medical students combining emotion recognition with extraversion received more positive evaluations. In particular, SPs were more satisfied with student’s communication when students were both good at recognizing nonverbally communicated emotions and scored high on extraversion. These effects resulted from brief interactions lasting no more than 5 min.

The question resulting from these findings is why SPs gave better evaluations to students with the same emotion recognition abilities when they scored high on extraversion [16]. We suggest that the SPs, similar to patients in interactions with their physicians, seek cognitive clarity and professional authority [34]. Extraverted physicians might create a positive atmosphere while at the same time taking the lead in diagnostic conversations, which patients might interpret as signals of expertise and, therefore, trustworthiness [46]. Expressing uncertainty in simulated communication situations was indeed shown to lead to a negative evaluation of medical students [47]. Physicians with more modest behavior might display compassion but not be as effective at inquiring about patients, collecting necessary information, and taking the lead, which patients might expect from them. Also, not all patients want to be part of the treatment decisions and seem to prefer physicians who take the lead in the decision-making process [48]. Patients aged 45 years and older especially like to leave the final decision to their physicians [48]. The median age of the SPs in this study was 59 years, so this could also be one reason why extraverted students combining high emotional recognition were rated higher for empathic communication.

With regard to the other four personality traits, this finding is surprising. In particular, people with more agreeableness are known for altruism and prosocial behavior and score high on self-assessed empathy [12, 49]. A possible explanation for not finding a correlation between agreeableness combined with high emotional recognition on SPs perceived empathic communication in this study could be the fact that the internal consistencies in the BFI-K questionnaire were low. Also, conscientiousness had a low internal consistency here, so that no reliable conclusions can be drawn for these two personality traits. On the other hand, communication from students scoring high on openness was rated less empathic. Although people showing high scores in openness are known to be very emotional, this finding is not unexpected [11]. Medical teachers displaying high levels of openness were evaluated as less adequate feedback-givers [35]. Verbal and non-verbal feedback towards the patients outlined problems is part of showing empathic care for a patient. Thus, less feedback could lead to the feeling of a lack of interest in patients concerns [50]. As for neuroticism, no positive or negative correlation with empathy has been reported [32]. Another explanation why extraversion was the only personality trait in this study which, in combination with emotion recognition, showed a significant increase of perceived empathic communication could be that extraverted people combine the motivation incentives of communion and agency [51]. This means that they not only show a desire to get in connection with other people and be a part of a union, they also have a motivation for achievement. On the other hand, agreeable people, for example, have only communal motives, which could be summarized as a need for affiliation and intimacy and miss a motivation for taking the lead [51].

If simulated patients prefer extraverted medical students scoring high in emotion recognition, the question arises as to why dispositional traits like the Big Five and emotional abilities are not assessed in combination in medical school admissions. One reason might be that self-assessments of personality traits also have drawbacks. Griffin and colleagues found that more than 60% of medical school applicants appeared to fake their personality tests. Successful and unsuccessful applicants exhibited similar scores in initial assessments but, when re-tested later, successful students demonstrated significantly lower scores in conscientiousness, extraversion, openness, and agreeableness, while also scoring higher for neuroticism when responding in non-evaluative contexts [52]. Translated to this study, however, this typical pattern of socially desirable responses seen in admission tests does not necessarily distort covariances between variables, as long as higher scoring leaves the ranking of respondent scores unchanged [53]. Another reason might be that personality measures failed to predict examinations outcomes: For example, McKenzie and colleagues analyzed the use of personality trait assessment forms for the prediction of exit performance [16]. These authors investigated more than 3000 students and could not show any predictive validity of self-report measures of personality traits on study performance [16].

Given the flaws in self-report measures of personality assessment, we, therefore, suggest that such measures might be combined with assessments using SPs. Simulated patients have been shown to be useful in other clinical examinations, particularly in assessing student empathy [23, 29]. The problem with such studies is often the lack of a methodologically sound dyadic-level data structure. Nevertheless, drawing on SPs might also make it possible to assess interrater reliability between SPs and clinical examiners. Accordingly, there is no reason not to use SPs for such examinations [29].

Our results also showed that male SPs gave significantly lower ratings for empathic communication than female SPs. These differences are interesting because gender seems to be an important factor not only on the side of the medical students but also from the side of the SPs. Other studies have also found significant differences between male and female SPs and patients, but the results are inconsistent. Borracci and colleagues found that, compared to women, male patients assign higher scores for empathic communication if rated by patients [54]. These authors argued that female patients seem to be more demanding of empathic communication [54]. Moreover, other studies investigated interactions between patients and physicians’ gender. Hall and coauthors show that male patients examined by young female physicians reported the lowest satisfaction in an investigation of medical visits [55]. In our case, we found no interaction between SPs and students gender, but in this OSCE setting, male SPs apparently rated more strictly than female SPs. This could be the same effect Schleicher and colleagues showed for examiners in OSCEs [56]. In this study, at five medical schools, female examiners rated student performance generally higher, whereas male examiners scored significantly higher if the examinee was female [56].

As a consequence of our study and the findings of other studies, we suggest that greater attention should be paid to personality development and training in emotion recognition skills during medical studies. The sole aim of medical school application tests should not be only to recruit students with strong cognitive abilities but also to develop their communication skills and empathy in doctor-patient relationships. Self-report personality tests could be applied not to select students but to identify their strengths and weaknesses and define their communication skills, which need development during medical school. Students might undergo communication training during medical school, raising their awareness of nonverbal signals in communication and coaching them appropriate responses. Several studies have shown that it is possible and necessary to improve communication skills and emotion recognition among medical students [17, 57]. Test settings, as demonstrated in this study, could help to identify students who need more training in emotion recognition and interpersonal communication.

### Limitations

This study has several limitations. First, this investigation focused on a relatively small group of students at one point in their medical training. Thus, this study might not reflect the performance of students in earlier or later years of medical school. We chose students during the middle of their medical education who had already had some experience with patients but who had not had daily patients interaction. Second, using self-assessment forms to estimate personality dimensions might have resulted in biased data. In particular, the internal consistency of agreeableness was rather low, which may have contributed to the non-significant results of the analyses.

The ICCs suggested that a significant proportion of the variance in perceived empathy resided on the level of SPs. Therefore, future research on the impact of patients’ personality on their evaluation of physicians’ performance is warranted. Thirdly, this study only used one approach to measure emotion recognition and perceived emphatic communication. Emotion recognition was only measured in static faces. Future research should replicate these results with more dynamic facial expressions and also include other channels of nonverbal and paralingual communication, such as body posture and tone of voice.

Despite these limitations, this study also has several strengths. First, we used a multilevel design, which made sure that every student was assessed by several SPs. Second, we can pinpoint the effects of students’ extraversion and emotion recognition ability on the dyadic level of interactions. Third, we avoided mono-method bias by combining self-report measures of extraversion, performance test measures for emotion recognition, and third-party ratings of satisfaction.

One might argue that the effect of emotion recognition and extraversion on patient satisfaction was small; however, this resulted from a brief interaction taking place under exam conditions and lasting no more than 5 min. Given this context, the findings lend support to the idea that social skills are influential even on first impressions in a doctor-patient relationship. Future research is therefore warranted to analyze the effects in timely unrestricted interactions between physicians and patients.

## Conclusions

The present results suggest that medical students who are both extraverted and good in emotion recognition give SPs a feeling of empathic communication. Therefore, students with an extraverted personality profile and more emotional abilities might seem to be more apt to fulfill the requirements of different medical roles as a doctor compared to other students. To date, student admission tests have been oblivious to this finding. Future doctors should be aware of this fact and train not only their knowledge but also their emotion recognition abilities. As a consequence of these results, we argue that medical students need to be trained in both emotion recognition and communication skills.

## Abbreviations

BFI: Big five inventory
CARE: Consultation and Relational Empathy scale
DANVA-2-AF: Diagnostic Analysis of Nonverbal Accuracy-2, Adult Facial Expressions
EI: Emotional intelligence
ER: Emotion recognition
ICC: Intraclass correlation
M: Mean
OSCE: Objective structured clinical examination
SD: Standard deviation
SE: Standard error
SP: Simulated patients
t: T-value
